# Coal with Carbon Capture and Sequestration is not as Land Use Efficient as Solar Photovoltaic Technology for Climate Neutral Electricity Production

**DOI:** 10.1038/s41598-018-31505-3

**Published:** 2018-09-07

**Authors:** James Gunnar Groesbeck, Joshua M. Pearce

**Affiliations:** 1 Department of Energy Engineering, Polytechnical University of València, Camino de Vera s/n, Valencia, 46022 Spain; 2 Department of Energy Technology, University of Stockholm (King’s Technical University) Brinnellvagen 68, 114 28 Stockholm, Sweden; 3 Department of Materials Science & Engineering, Michigan Technological University, 601 M&M Building, Houghton, MI 49931-1295 USA; 4 Department of Electrical & Computer Engineering, Michigan Technological University, 601 M&M Building, Houghton, MI 49931-1295 USA; 5 Department of Electronics and Nanoengineering, School of Electrical Engineering, Aalto University, 02150 Espoo, Finland

## Abstract

Avoiding climate destabilization caused by greenhouse gas (GHG) emissions, requires climate-neutral electricity sources. It has been proposed that the GHG emissions from coal-fired power plants can be offset by carbon capture and sequestration or bio-sequestration. However, solar photovoltaic (PV) technology has recently declined so far in costs it now offers both technical and economic potential to offset all of coal-fired electricity use. PV only emits GHGs during fabrication and not during use. To determine which technical solution to climate-neutral electricity generation should be preferred, this study aggregates and synthesizes life cycle analysis studies for exergy, GHG emissions and land transformation for climate-neutral electricity. The results show that because of lower exergy efficiencies coal plants emit 13–18 times more GHG and transform 5–13 times more land than PV. Optimal bio-sequestration of coal-fired GHG requires 62% of U.S. arable land or 89% of all U.S land with average forest cover. Carbon capture and storage and enhanced oil recovery can improve coal performance, but for all cases the results clearly show that PV is a far more effective use of land. Overall, for the first time this study found climate-neutral photovoltaic farms are a preferred solution to climate-neutral coal fired electricity generation.

## Introduction

It is now well established that global climate change is underway because of greenhouse gas (GHG) emissions dominated by anthropogenic energy production^1^. This has negative impacts on natural and socio-economic systems^2,3^. GHG emissions increase global temperatures^4^, which in turn increase sea levels^5^, extinction rates among animals^6^ and also harms human health^7,8^ and the stability of traditional power generation^9^. GHG emissions are dominated by carbon dioxide (CO_2_)^10^ with 40% of CO_2_ emissions coming from traditional electrical power generation^11^. There is a clear need to mitigate climate change by reducing emissions during energy generation^12,13^. This can be accomplished in part through the use of climate-neutral renewable and traditional power generation^14–17^.

Climate-neutral electricity generation, where the life cycle CO_2_ equivalent of all GHG emissions from an energy source are eliminated, would have the largest single potential benefit to mitigating climate change in the future as transportation moves toward electrification. Although, selecting a climate-neutral power source is challenging, the concept of exergy can be used to guide decision making. Exergy can be thought of as the useful energy available and is advantageous for comparing systems with different grades of energy^17^. Energy efficiencies are misleading because they lack proper accounting for sources of waste heat and irreversibility. For example, low-temperature heat from solar thermal collectors is less useful than electricity from solar photovoltaic (PV) systems, although PV efficiencies are less than that of solar thermal systems^18^. Energy cannot be lost, while in real systems these irreversible entropy losses are quantified by exergy efficiency^19,20^.

The largest producer of electricity is coal^21^ whose CO_2eq_ emissions demand some form of carbon capture and sequestration (CCS) in order to be climate neutral. Typically, CCS is the capture and separation of CO_2_ and subsequent compression and transport to storage locations such as saline aquifers^22,23^. A popular form of CCS utilizes enhanced oil recovery (EOR), which pumps CO_2_ into an operational oil and gas reservoir to displace more oil and gas^22,24^. However, another form of CCS is the planting of biomass to permanently absorb and store carbon either in itself or the soil^25^, referred to here as bio-sequestration. All of these processes have their own downstream emissions and coupled with the remainder of life cycle emissions, climate neutral coal-fired electricity generation requires large areas of land, especially for bio-sequestration^26^.

On the other hand, solar PV has the greatest potential to scale to provide for sustainable future among renewable sources^27^, but demands large surface areas during operation^28^. PV also has embodied energy, which results in upstream emissions. So climate-neutral PV also requires land transformation for bio-sequestration.

**Figure 1:**
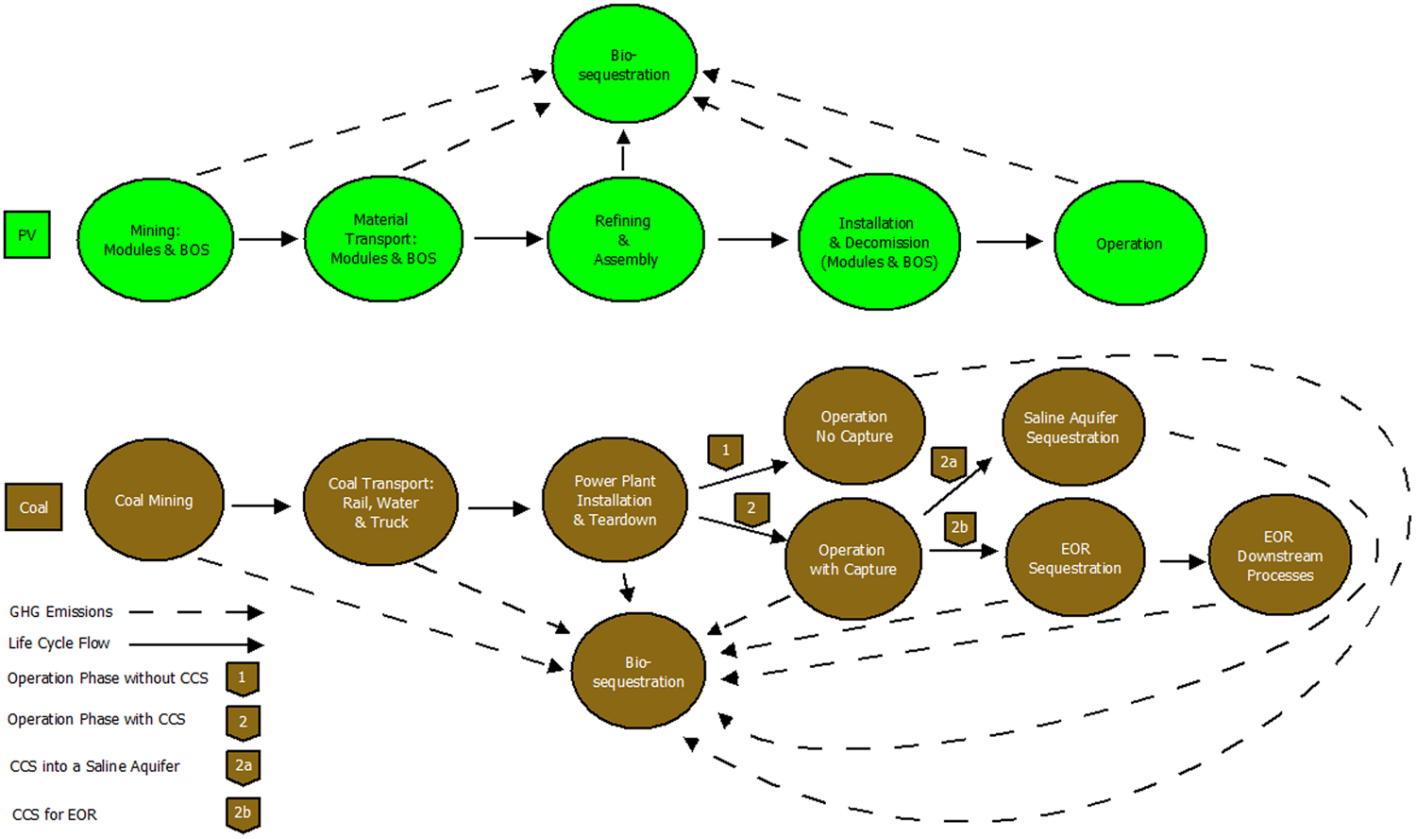
LCEA boundary scope for climate-neutral PV and pulverized coal electricity production. The solid arrows represent the flow of the life cycle, the dashed lines represent the CO_2eq_ of the GHG emissions uptake by bio-sequestration and the labels detail various the scenarios for the coal lifecycle.

The purpose of this study is to determine if is a better use of land and energy to produce climate neutral electricity with coal (and some form of carbon sequestration) or PV. Several life cycle analysis (LCA) studies are aggregated here to determine the preferred approach to climate neutral electricity generation. This study compares exergy, GHG emissions and land transformation needed for climate-neutral solar PV and climate-neutral pulverized coal with and without utilizing various forms of CCS. The climate-neutral status of a given technology is attained through a combination of bio-sequestration and CCS in saline aquifers or oil and gas reservoirs during EOR. PV and coal-based climate neutral energy solutions are analyzed using power plants with equivalent lifetime electricity output in a complete comparative analysis using aspects of exergy analysis and LCA’s, summarized in Fig. 1.

## Climate-Neutral Coal Plants

Combustion of coal for electricity produces CO_2_ directly and a method is needed to eliminate the effect of these emissions on the atmosphere and the climate. The coal plant analyzed has a 1GW nameplate capacity and a capacity factor of 85%, which produce 376 TWhrs over a 50 year lifetime. The natural environment has a substantial capacity to store carbon near permanently, referred to here as bio-sequestration^26^, but they are land area intensive so several methods are analyzed to reduce this impact from coal.

The most common process to capture CO_2_ from a coal plant uses monoethanolamine (MEA) post-combustion for flue gas separation. Membranes with one and two step sweeps, pre-combustion gasification, oxidation, solid sorbents, metal organic frameworks, diethanolamine (DEA) and methyldiethanolamine (MDEA) and others have been developed as well to separate CO_2_ from coal flue gases. In order to be included in this analysis, at least 80% of the carbon had to be captured. These processes are energy intensive which derates electrical generation of a coal plant^29^. In order to maintain the same output, it is assumed that more coal is combusted to offset the drop in efficiency with the addition of CCS. The captured CO_2_ is compressed to a supercritical state, typically between 8.6–15.3 MPa, and transported through pipelines to the storage location^30^. In order to theoretically offset the GHG emissions from the coal lifecycle, both with and without CCS, bio-sequestration is employed here, specifically switchgrass as it has the best rate of carbon uptake and sequestration potential^26^.

Attempting to mimic natural carbon sequestration, CCS has received a lot of attention recently^31^. Globally, potential CO_2_ storage in geological formations is between 200–2,000 Gt, with saline aquifers comprising the majority^32^. There are currently 16 active CCS projects globally, injecting 30.15 ${{\rm{t}}}_{{{\rm{CO}}}_{{\rm{2}}}}$/yr with another 22 projects planned for the next 10–15 years^32^. Once CO_2_ is sequestered in geological formations like saline aquifers and oil and gas reservoirs, it has to be monitored to quantify leakage^33^, which also must be offset for climate neutrality. 13 of the 16 active projects employ EOR to partially or fully sequester the CO_2_. However, as EOR can increase the productivity of an oil reservoir from 25–55% to 35–75%^34^, the additional exergy output and CO_2_ emissions from downstream processes like oil refining and combustion must be accounted for, Which in turn demands further land transformation for bio-sequestration. The net exergy output of coal with CCS for EOR is 866 TWhrs over its lifetime.

## Climate-Neutral Photovoltaic Farms

To compare directly to the coal plants, two fixed-panel solar PV farms are designed to similarly produce 376 TWhrs (PVs1) and 866 TWhrs (PVs2) over a 50 year lifetime^35^ with a degradation rate of 0.49%/yr^36,37^. Assuming a capacity factor of 18.3% and performance ratio of 0.89, this results in a nameplate capacity of 5.23 GW for PVs1 and 12.13 GW for PVs2. The embodied emissions from the PV farm can be roughly broken down to three main categories, modules, balance of system (BOS) and construction/decommission^38^. The values for exergy, emissions and land transformation include the impacts from extraction of raw materials, transportation, refining to solar grade silicon, assembly of modules, construction of the PV farm and implementation of bio-sequestration for all emissions with switchgrass.

## Results: Comparison of Exergy, Emissions and Land Transformation in 1GW-Equivalent Climate Neutral Photovoltaic and Coal Power Plants

This study compares the exergy, GHG emissions and land transformation needed for climate-neutral pulverized coal with and without utilizing various forms of CCS and climate-neutral solar PV.

### Climate-neutral coal plants

Three scenarios for carbon sequestration are analyzed: 1) no carbon capture technology at the plant, and instead uses bio-sequestration to uptake the carbon entirely; and plant level carbon capture of at least 80% and pipe it to 2a) a saline aquifer with remaining emissions using bio-sequestration, or 2b) for use in EOR, with all remaining emissions using bio-sequestration.

The coal plant analyzed here has individual contributions to upstream activities from mining and transport of coal and the construction/decommission of the coal plant. Mining and transport account for the majority of exergy input emissions and land transformation with the tonnage of coal consumed by the plant being the main driver. Upstream activities of a coal plant without carbon capture requires 108 TWhrs of exergy input, emits 3.92 × 10^7^ ${{\rm{t}}}_{{{\rm{CO}}}_{2}}$_eq_ and transforms 17.8 kha of land for bio-sequestration. The addition of carbon capture technology pushes this to require 149 TWhrs, GHG emissions of 5.34 × 10^7^ ${{\rm{t}}}_{{{\rm{CO}}}_{2}}$_eq_ and transforms 22.7 kha^39,40^. The carbon capture option requires more coal due to the lower efficiency plant.

During the operation of the coal plant, the effects of adding carbon capture technologies is studied. Additional coal input is used to offset the derating of the plant due to carbon capture to ensure a 1GW nameplate capacity. A typical state-of-the-art plant drops from an efficiency of 37% to 27% with the addition of carbon capture technology^22,29,36,41,42^. If the upstream exergy input is subtracted from the exergy output, then the net efficiency drops to 27% without carbon capture and 17% with carbon capture. In power plants without carbon capture, 995 TWhr_eq_ of coal is required, resulting in 1.52 × 10^8^ t_coal_. GHG emissions total to 3.38 × 10^8^ ${{\rm{t}}}_{{{\rm{CO}}}_{2}}$_eq._^39,43^. The total land required is 361 kha, with bio-sequestration transforming 343 kha and the plant alone transforms 202 ha^26,38,40^. The physical area required for the plant is considered constant with and without CCS.

In power plants with CCS, the exergy input from coal equates to 1370 TWhrs and emits 6.07 × 10^7^ ${{\rm{t}}}_{{{\rm{CO}}}_{2}}$_eq_ to the atmosphere^44,45^. The total GHG emissions produced from both upstream and during operation are 4.52 × 10^8^ ${{\rm{t}}}_{{{\rm{CO}}}_{2}}$ with 3.38 × 10^8^ ${{\rm{t}}}_{{{\rm{CO}}}_{2}}$ going to storage. If the captured CO_2_ is piped to a saline aquifer for sequestration, this results in the uptake of 1.18 × 10^8^ ${{\rm{t}}}_{{{\rm{CO}}}_{2}}$ that were released to the atmosphere for bio-sequestration^38,44,45^. The slight discrepancy in uptake by bio-sequestration is from the emissions due to leakage. The total land transformation is 132 kha, with bio-sequestration requiring 109 kha alone^9,26,31,38,40^.

In power plants with CCS for EOR, the subsequent downstream activities require 1400 TWh’s of exergy input, which totals to 2.16 × 10^8^ GWhrs and emit an additional 1.93 × 10^8^ ${{\rm{t}}}_{{{\rm{CO}}}_{2}}$_eq_ to the atmosphere. This means that 3.11 × 10^8^ ${{\rm{t}}}_{{{\rm{CO}}}_{2}}$_eq_ will need to be bio-sequestered^32,38,39,43–45^, which necessitates 307 kha of total land transformation with 284 kha for bio-sequestration^9,26,31,38,40,43,46^.

The downstream processes for EOR also produce additional exergy output. In order to give a direct comparison to PV, the exergy output from EOR-based refined product is combusted with an efficiency of 39%^47^ to generate electricity. The total lifetime exergy output for the EOR scenario becomes 866 TWhrs net electricity. A more in depth breakdown of the exergy flow, emissions and land transformation for coal with various forms of CCS can be found in Tables 1 and 2.

**Table 1:** Overview of exergy flow, emissions and land transformation by life cycle phase in a climate neutral coal plant outputting 376 TWhrs of electricity over a 50 year lifetime.

Life Cycle Phase	Source/Sink	Exergy_in_ (GWh)	Exergy_out_ (GWh)	Emissions* (${{\bf{t}}}_{{\bf{C}}{{\bf{O}}}_{{\bf{2}}}}$_eq_)	Land Transformation (ha)
Upstream without CCS	Mining	6.69 × 10^4^ ^22,29,36,42,56–60,62^		2.16 × 10^7^ ^39,43^	1.32 × 10^4^ ^40,48,64^
	Transport	3.17 × 10^4^ ^39^		1.76 × 10^7^ ^39,43^	4.32 × 10^3^ ^39,48,76^
	Construction	1.29 × 10^4^ ^39,63^		1.66 × 10^5^ ^39,63^	N/A
	Total	1.11 × 10^5^ ^22,29,36,39,42,56–60,62^		3.92 × 10^7^ ^39,43,63^	1.78 × 10^4^ ^3,40,48,64,76^
Upstream with CCS	Mining	9.21 × 10^4^ ^22,29,42,56–61^		2.94 × 10^7^ ^39,43^	1.82 × 10^4^ ^40,48,64^
	Transport	4.37 × 10^4^ ^39^		2.40 × 10^7^ ^39,43^	4.32 × 10^3^ ^39,48,66^
	Construction	1.29 × 10^4^ ^39,63^		1.66 × 10^7^ ^39,63^	N/A
	Total	1.49 × 10^5^ ^22,29,39,42,56–61,63^		5.34 × 10^7^ ^39,43,63^	2.27 × 10^4^ ^39,40,48,64,66^
Operation without CCS	Plant	9.95 × 10^5^ ^22,29,36,42,57,60,62^	3.76 × 10^5^	3.38 × 10^8^ ^38,44,45,58^	2.02 × 10^2^ ^48^
Operation with CCS	Plant	1.37 × 10^6^ ^22,29,41,42,57,59–61^	3.76 × 10^5^	6.07 × 10^7^ ^22,38,54,45,58^	2.02 × 10^2^ ^48^
Downstream without CCS	Bio-sequestration	2.57 × 10^8^ ^38,39,43–45,53,58,79,83^		−3.77 × 10^8^ ^38,39,43–45,58,63^	3.43 × 10^5^ ^38,39,43–45,53,58,63,74^
Downstream with CCS into a saline aquifer	Bio-sequestration	8.14 × 10^7^ ^10,22,31,32,38,39,42–45,53,58,63,79,83,96^		−1.18 × 10^8^ ^10,22,31,32,38,39,42–45,53,58,63,79,96^	1.09 × 10^5^ ^10,22,31,32,38,39,42–45,53,58,63,79,83,96^
	CO_2_ Conditioning	2.64 × 10^4^ ^41,79^		1.97 × 10^6^ ^10,32^	N/A
	CO_2_ Injection	1.57 × 10^3^ ^43,78^		N/A	N/A
	CO_2_ Leakage	N/A		3.53 × 10^6^ ^31,82^	N/A

*Carbon sequestration as negative and carbon equivalent emissions as a positive numbers.

**Table 2:** Overview of exergy flow, emissions and land transformation by life cycle phase in a climate neutral coal plant outputting 376 TWhrs and utilizing EOR for an additional output of 491 TWhrs of electricity over the 50 year lifetime, totaling 866 TWhrs.

Life Cycle Phase	Source/Sink	Exergy_in_ (GWh)	Exergy_out_ (GWh)	Emissions* (${{\bf{t}}}_{{\bf{C}}{{\bf{O}}}_{{\bf{2}}}}$_eq_)	Land Transformation (ha)
Upstream with CCS	Mining	9.21 × 10^4^ ^22,29,42,56–61^		2.94 × 10^7^ ^39,64^	1.82 × 10^4^ ^40,48,64^
	Transport	4.37 × 10^4^ ^39^		2.40 × 10^7^ ^39,64^	4.32 × 10^3^ ^39,48,66^
	Construction	1.29 × 10^4^ ^39,63^		1.66 × 10^7^ ^39,63^	N/A
	Total	1.49 × 10^5^ ^22,29,39,42,56–61,63^		5.34 × 10^7^ ^39,43,63^	2.27 × 10^4^ ^39,40,48,64,66^
Operation with CCS	Plant	1.37 × 10^6^ ^22,29,42,57,59–61^	3.76 × 10^5^	6.07 × 10^7^ ^22,38,44,45,58^	2.02 × 10^2^ ^48^
Downstream with CCS for EOR	Bio-sequestration	2.13 × 10^8^ ^10,22,31,32,34,38,39,42–46,53,58,63,78–81,83,96,97^		−3.11 × 10^8^ ^10,22,31,32,38,39,42–46,53,58,63,78,79,96^	2.84 × 10^5^ ^10,22,31,32,38,39,42–46,53,58,63,78,79,83,96^
	CO_2_ Conditioning	2.64 × 10^4^ ^41,79^		1.97 × 10^6^ ^10,32^	N/A
	Crude Oil Extraction	8.76 × 10^3^ ^22,34,42,44–46,58,61,78,81,97^		3.51 × 10^7^ ^22,42,44–46,58^	N/A
	CO_2_ Injection/Recycling	1.57 × 10^3^ ^22,34,42,44–46,58,61,78,81,97^		3.87 × 10^6^ ^22,42,44–46,58,78^	N/A
	Crude Oil Transport	1.86 × 10^4^ ^22,34,42,44–46,58,78,81,97^		1.35 × 10^6^ ^22,42,44–46,58,78^	N/A
	Crude Oil Refining	1.38 × 10^6^ ^22,34,42,44–46,58,78,80,81,97^		1.02 × 10^7^ ^22,42,44–46,58,78^	N/A
	Petroleum Combustion	N/A	4.91 × 10^5^	1.46 × 10^8^ ^22,42,44–46,58,78^	N/A
	CO_2_ Leakage	N/A		3.53 × 10^6^ ^31,82,98^	N/A

*Carbon sequestration as negative and carbon equivalent emissions as a positive numbers.

### Climate-neutral solar photovoltaic farms

The upstream exergy input, emissions and land transformation can be separated into three categories, modules, BOS and construction of the PV farm. The total exergy input is 20.0 TWhrs. The majority of GHG emissions occur upstream, totaling 8.92 × 10^6^ ${{\rm{t}}}_{{{\rm{CO}}}_{2}}$_eq_ and transforms 584 ha of land for bio-sequestration and the PV farm physical footprint^38,39,48–50^.

The desired electrical output for PVs1 is 376 TWhrs over the 50 year lifetime. The exergy efficiency of the PV system is 12.1%^51,52^, which is rather conservative, and together with the U.S. average solar irradiation of 15,000 GWh/ha-yr it provides the required exergy input of 4,330 TWhrs. Moreover, 8.69 × 10^4^ ${{\rm{t}}}_{{{\rm{CO}}}_{2}}$ are emitted from the location of the PV farm from vegetation clearing and soil respiration^38^. A key driver of land transformation in the life cycle of PV is the farm itself, transforming 3.94 kha of land^38,48^.

The exergy input from solar irradiation for bio-sequestration is 12,900 TWhrs. Total GHG emissions to be offset with bio-sequestration are 9.01 × 10^6^ ${{\rm{t}}}_{{{\rm{CO}}}_{2}}$_eq_, transforming 17.2 kha of land^26,38,49,50^.

Finally, in order to give an accurate comparison of PV to coal with EOR, it is necessary to maintain an equivalent exergy output, so a second scenario with an electrical output of 866 TWhrs over the 50 year lifetime is employed, referred to here as PVs2. All exergy inputs, GHG emissions and land transformation has been scaled up ~2.3 to reflect the larger output. More in depth information on PVs1 and PVs2 can be found in subsequent sections and Tables 3 and 4.

**Table 3:** Overview of exergy flow, emissions and land transformation by life cycle phase in a climate neutral PV farm outputting 376 TWhrs of electricity over the 50 year lifetime.

Life Cycle Phase	Source/Sink	Exergy_in_ (GWh)	Exergy_out_ (GWh)	Emissions* (${{\bf{t}}}_{{\bf{C}}{{\bf{O}}}_{{\bf{2}}}}$_eq_)	Land Transformation (ha)
Upstream	Modules	1.25 × 10^4^ ^49,50,67^		3.47 × 10^6^ ^38,49,50,67–69^	4.15 × 10^2^ ^48^
	BOS	7.36 × 10^3^ ^49,50,67^		5.45 × 10^6^ ^38,49,50,67–69^	1.69 × 10^2^ ^48^
	Construction	7.16 × 10^1^ ^39,63^		2.52 × 10^5^ ^39,63^	N/A
	Total	2.00 × 10^4^ ^39,49,50,63^		8.92 × 10^6^ ^38,49,50,68,69^	5.84 × 10^2^ ^48^
Operation	Farm	3.50 × 10^4^ ^37,51,52,70,71,73^	3.76 × 10^5^	8.69 × 10^4^ ^38^	9.51 × 10^3^ ^38,48,74–76^
CCS	Bio-Sequestration	1.29 × 10^7^ ^38,49,50,53,68,69,79,83^		−9.01 × 10^6^ ^38,49,50,68,69^	1.72 × 10^4^ ^38,49,50,68,69,83^

*Carbon sequestration as negative and carbon equivalent emissions as a positive numbers.

**Table 4:** Overview of exergy flow, emissions and land transformation by life cycle phase in a climate neutral PV farm outputting 866 TWhrs of electricity over the 50 year lifetime.

Life Cycle Phase	Source/Sink	Exergy_in_ (GWh)	Exergy_out_ (GWh)	Emissions* (${{\bf{t}}}_{{\bf{C}}{{\bf{O}}}_{{\bf{2}}}}$_eq_)	Land Transformation (ha)
Upstream	Modules	2.89 × 10^4^ ^49,50,67^		6.62 × 10^6^ ^38,49,50,67–69^	7.97 × 10^2^ ^48^
	BOS	1.70 × 10^4^ ^49,50,67^		1.04 × 10^7^ ^38,49,50,67–69^	3.25 × 10^2^ ^48^
	Construction	1.43 × 10^2^ ^39,63^		5.81 × 10^5^ ^39,63^	N/A
	Total	4.60 × 10^4^ ^39,49,50,63^		1.70 × 10^7^ ^38,49,50,68,69^	1.12 × 10^3^ ^48^
Operation	Farm	8.05 × 10^7^ ^37,51,52,70,71,73^	8.66 × 10^5^	2.01 × 10^6^ ^38^	2.65 × 10^4^ ^38,48,74–76^
CCS	Bio-sequestration	2.59 × 10^7^ ^38,49,50,53,68,69,79,83^		−1.72 × 10^7^ ^38,49,50,68,69^	3.45 × 10^4^ ^38,49,50,68,69,83^

*Carbon sequestration as negative and carbon equivalent emissions as a positive numbers.

### Exergy

**Figure 2:**
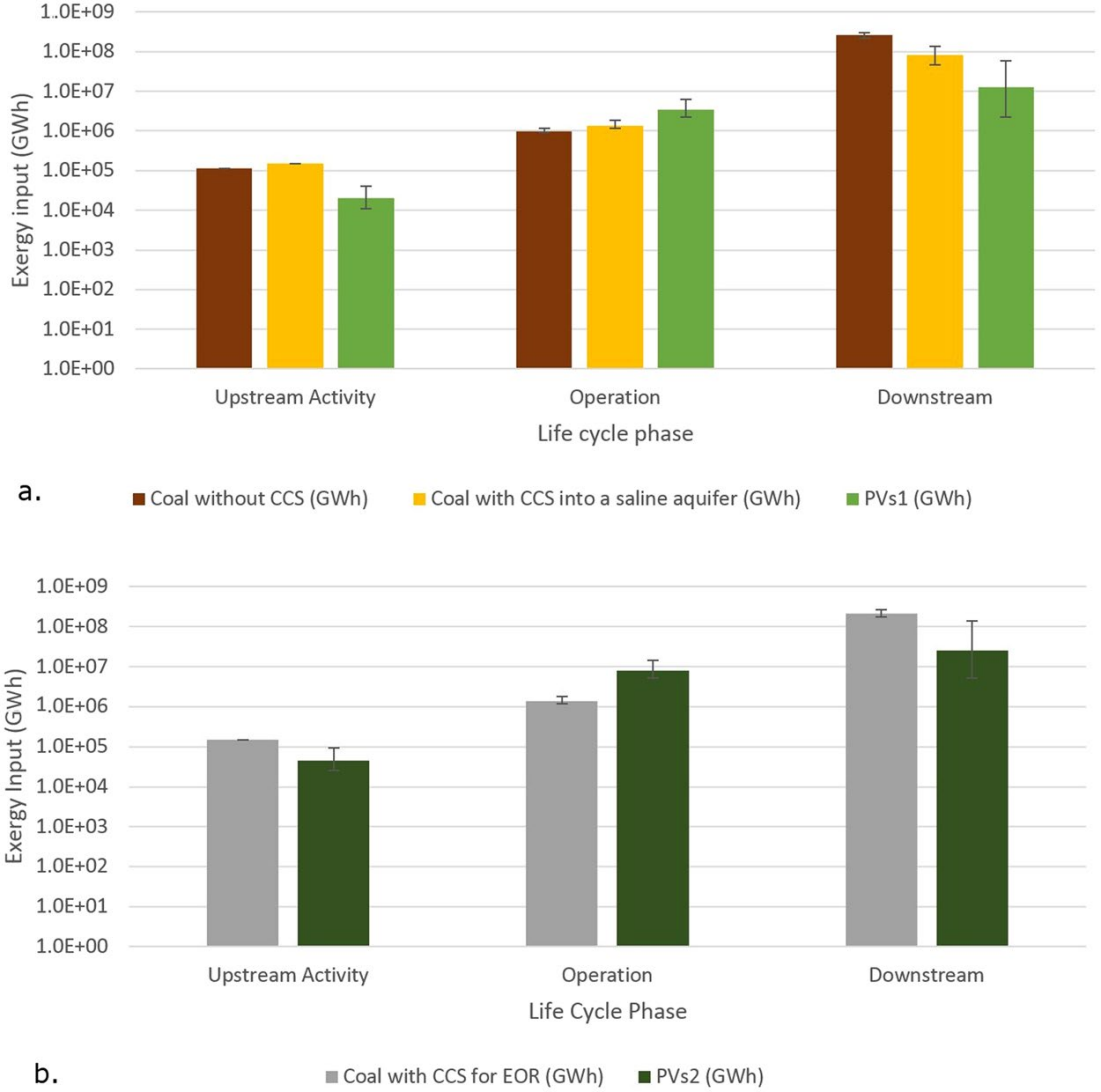
(**a**) Lifetime exergy input by life cycle phase comparing coal with and without capture into a saline aquifer and photovoltaics, each outputting 376 TWhrs. (**b**) Lifetime exergy input by life cycle phase comparing climate-neutral coal with EOR and climate-neutral photovoltaic plants, outputting 866 TWhrs. Error bars indicate boundary values.

The exergy analysis includes the inputs from solar irradiation and the heat content of coal, as well as electricity, diesel and various other sources used to produce electricity with the two methods. With these factored in, the exergy required from solar irradiation for bio-sequestration is orders of magnitude larger than the inputs for the other phases of the life cycle, as shown in Fig. 2a.

For upstream anthropogenic exergy inputs of coal and PV outputting 376 TWhrs, climate neutral coal requires between 92–129 TWhrs more, with a realistic value of 108 TWhrs more (3.24 to 7.45 times more than PVs2 and PVs1, respectively) than climate neutral PV, which results in 36–45 MMTCO_2_ with a realistic value of 40 MMTCO_2eq_ more emitted.

The additional coal required to offset the energy requirements of the CCS systems necessitates a larger exergy input, 30–38% with a realistic value of 33% more for upstream activities and a range of 31–58% with a realistic value of 38% more coal energy for plant operation. CCS reduces the solar exergy required for bio-sequestration, 2.47–4.69 with a realistic value of 3.16 times less if into a saline aquifer and 1.20–1.25 with a realistic value of 1.22 times less if for EOR than without CCS, illustrated in Fig. 2a and b.

The increased exergy input to upstream and operational phases for coal with CCS is negated by the solar exergy required for bio-sequestration. The error bars represent the minimum and maximum values found in literature for the life cycle phase of each scenario (e.g. these are boundary values not probabilistic estimates). In general, PV has a larger range of values, presumably because of the rapid rate of improvements in technology compared to coal.

### GHG Emissions

**Figure 3:**
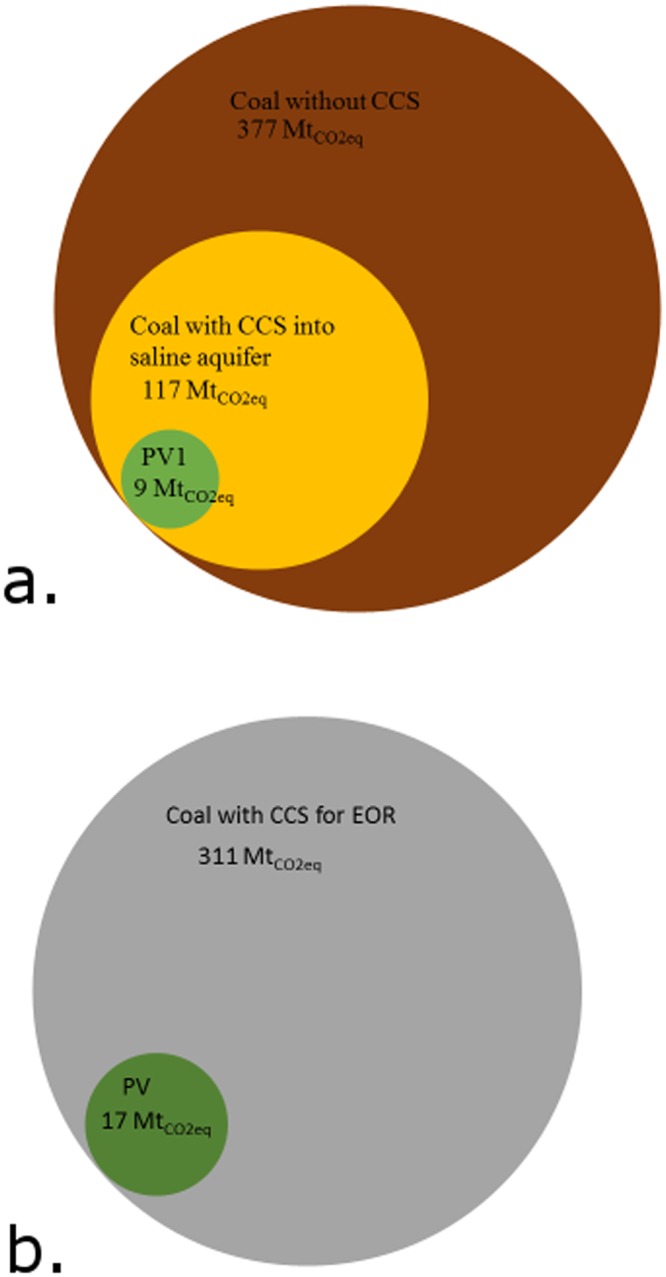
(**a**) Comparing LCA GHG emissions from a coal plant without carbon capture, a coal plant with saline aquifer CCS, and PVs1 farm. All use bio-sequestration to fully or partially sequester CO_2_ and all output 376 TWhrs of electricity. (**b**) Total LCA GHG emissions from a coal plant with CCS for EOR, and PVs2 farm. Both use bio-sequestration to fully or partially sequester CO_2_ and both net output 866 TWhrs of electricity over their lifetime.

Over a 50 year lifetime^36,37^, a photovoltaic farm outputting 376 TWhrs will emit 9.01 M${{\rm{t}}}_{{{\rm{CO}}}_{2}}$_eq_ ± 41.79/7.04 M${{\rm{t}}}_{{{\rm{CO}}}_{2}}$_eq_ to the atmosphere^38,49,50^, while coal with CCS into a saline aquifer emits 117.61 M${{\rm{t}}}_{{{\rm{CO}}}_{2}}$_eq_ ± 51.29/49.83 (over 13x more) and 377.11 M${{\rm{t}}}_{{{\rm{CO}}}_{2}}$_eq_ ± 98.48/58.06 M${{\rm{t}}}_{{{\rm{CO}}}_{2}}$_eq_ (over 41x more) without CCS, as seen in Fig. 3a ^22,29,36,39,43^. When the coal plant under study utilizes enhanced oil recovery to sequester CO_2_ emissions, additional crude oil is produced and is assumed to be combusted for electricity generation, totaling 866 TWhrs. It will emit 310.55 M${{\rm{t}}}_{{{\rm{CO}}}_{2}}$_eq_ ± 72.80/37.31 M${{\rm{t}}}_{{{\rm{CO}}}_{2}}$_eq._^29,36,39,43^. If the PV farm output is increased to match the net output from the coal plant with EOR, it produces 17.23 M${{\rm{t}}}_{{{\rm{CO}}}_{2}}$_eq_ ± 83.79/13.45 M${{\rm{t}}}_{{{\rm{CO}}}_{2}}$_eq_ (over 18x less) greenhouse gas emissions, as seen in Fig. 3b ^38,49,50^.

**Figure 4:**
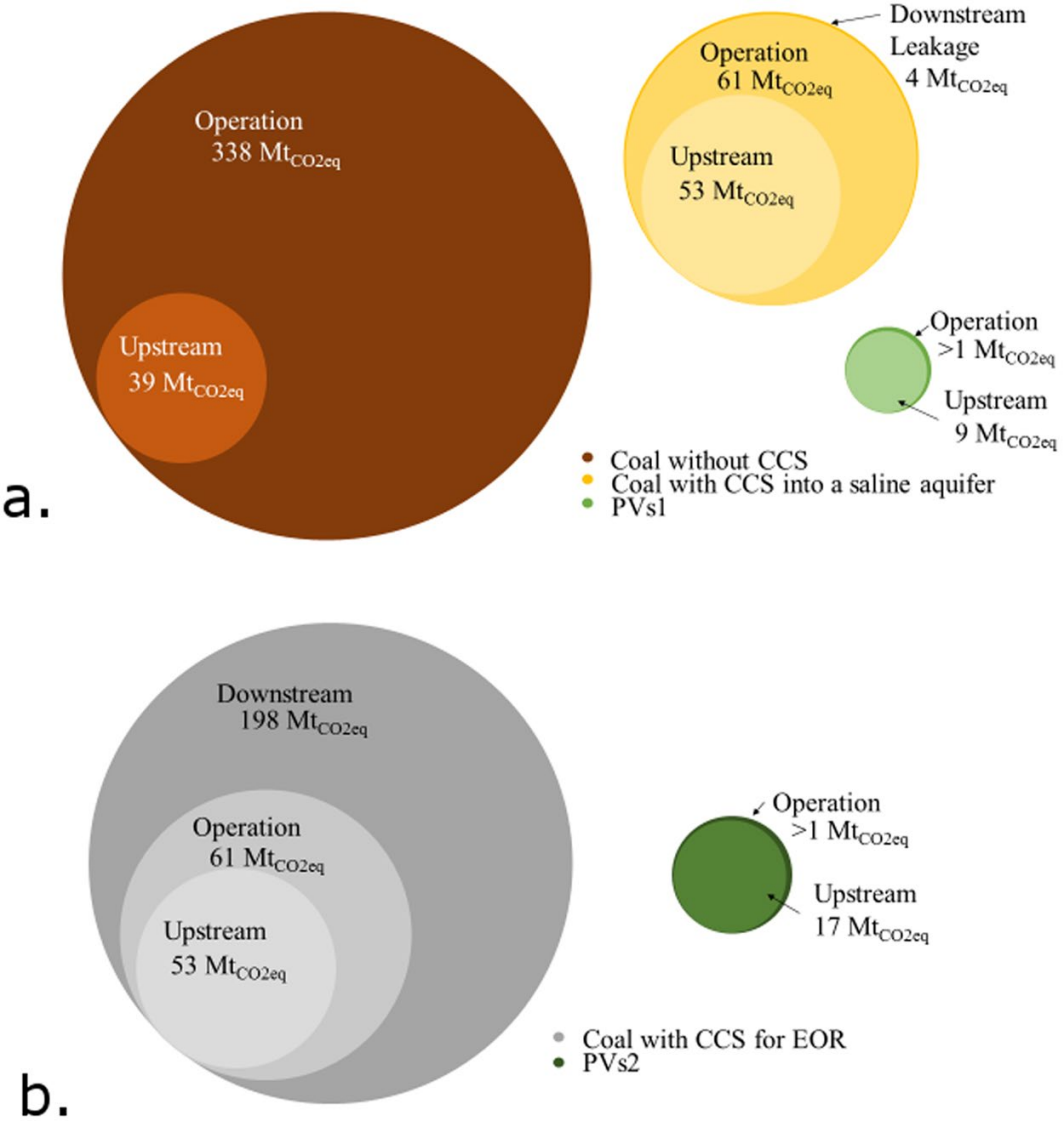
(**a**) To-scale visualization of GHG emissions by life cycle phase for coal without CCS, coal with CCS into a saline aquifer and PV, each outputting 376 TWhrs of electricity over their lifetimes. (**b**) To-scale visualization of GHG emissions by life cycle phase for, coal with CCS for EOR and PV, each outputting 866 TWhrs of electricity over their lifetimes.

The additional coal required to offset the derating of the plant due to carbon capture technology is made up for by a significant decrease in emissions released to the atmosphere during operation, as seen in Fig. 4a. The leakage of emissions after storage does not greatly affect the total, but there has been little public research on this for large-scale storage. The 2005 IPCC special report provided targets of 0.001%/yr to 0.01% per year. The EPA released regulations in 2011 for CCS leakage mitigation and monitoring stipulating zero leakage^33,53^, which has prompted companies to report zero leakage and hindered efforts for more accurate studies.

The combustion of refined oil product is less polluting than coal, which helps curtail the downstream emissions, but compared to PVs2 it is still significantly more polluting, as seen in Fig. 4b.

On a per GWh_out_ basis, coal without CCS, coal with CCS into a saline aquifer, coal with CCS for EOR and PV each emit 1004.23 ${{\rm{t}}}_{{{\rm{CO}}}_{2}}$_eq_/GWh_out_, 313.17 ${{\rm{t}}}_{{{\rm{CO}}}_{2}}$_eq_/GWh_out_, 358.56 ${{\rm{t}}}_{{{\rm{CO}}}_{2}}$_eq_/GWh_out_ and 23.99 ${{\rm{t}}}_{{{\rm{CO}}}_{2}}$_eq_/GWh_out_, respectively.

### Land Transformation

**Figure 5:**
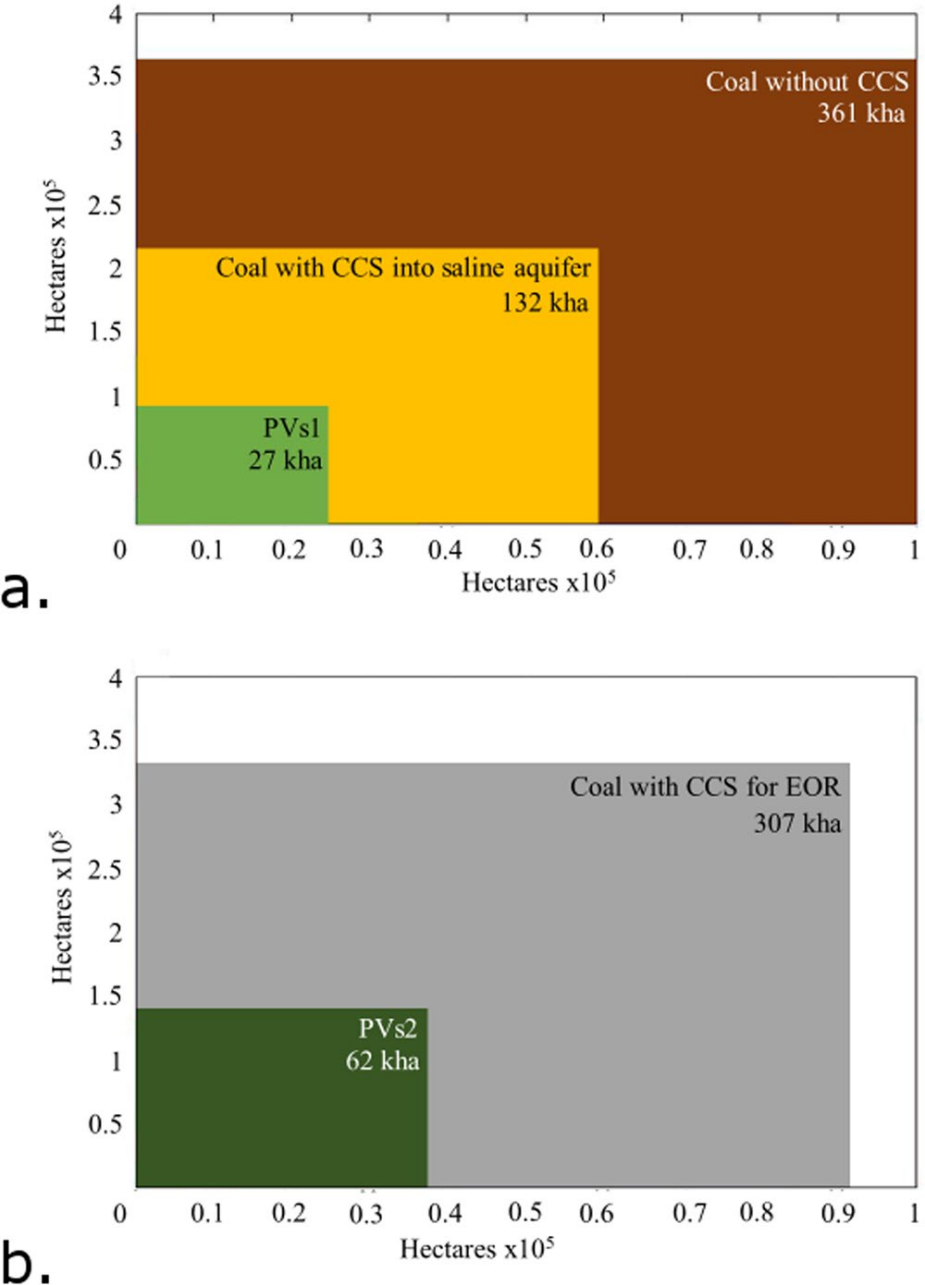
(**a**) Land transformation shown to scale in hectares for bio-sequestration required to provide for climate-neutral coal without CCS, coal with CCS into a saline aquifer and PV, each producing 376 TWhrs electricity over their lifetime. (**b**) Land transformation shown to scale in hectares for bio-sequestration required to provide for climate-neutral coal with CCS for EOR and PV, both producing 866 TWhrs electricity over their lifetime.

The amount of land transformed by equivalently sized climate-neutral PV electrical power generation is over 13x less than for climate-neutral coal electrical power generation. The use of CCS into a saline aquifer for climate-neutral coal plants helps reduce emissions to the atmosphere and drops it to 5x more than PV, as seen in Fig. 5a. Climate-neutral coal with CCS for EOR also requires 5x more land transformation because the increase in electrical production is offset by the combustion of oil, as seen in Fig. 5b.

The majority of land transformation for both PV and coal is for bio-sequestration. For all coal scenarios, 81–95% of the total land transformation is for bio-sequestration, while it is 76% and 78% for PVs1 and PVs2, respectively. For reference, 343 kha of land is transformed in the scenario for a single 1 GW power plant using coal without CCS, which is larger than the state of Rhode Island. If all emissions from coal-fired electricity power generation in the United States were bio-sequestered with switchgrass, it would require 62% of the arable land in the U.S^54^. With CCS into a saline aquifer, it would still require 20% of the arable land in the U.S. to be planted with switchgrass to bio-sequester the whole fleet^54^.

If the bio-sequestration were left to be performed by the less-efficient average forest in the U.S., then 8.5x more land would be required^55^, resulting in a new forest occupying an area larger than the state of Maryland for a 1GW coal plant without CCS. To bio-sequester the whole fleet of coal plants then a new forest would have to be 2.66 times larger than the existing forest in the U.S., which amounts to 88.54% of the area of the entire U.S.^54^. If CCS into a saline aquifer were utilized, a new forest with an area that is 85.77% of the existing forest in the United States is required^54^. It should be pointed out, however, that land use for biosequestration can have other applications (e.g. forests can be used for wildlife preservation and human recreation) whereas the land area specifically made up for CCS and PV generally can not be used for other applications.

## Methods

This section describes in detail the methods used to calculate the exergy, GHG emission and land transformation for coal and PV generation of electricity. When possible, several sources with data on state-of-the-art technology were used and minimum, maximum and average values were determined. The term *realistic* is used to describe the average value or a readily obtained technological value. The equations used to determine the values for exergy, GHG emissions and land transformation for all PV and coal scenarios are stated. SimaPro V8 was utilized and all exergy data is from Cumulative Exergy Demand V1.03 and GHG emissions data is from IPCC GWP 100a. Emissions from the electrical grid are not included in the scope of the LCEA’s for PV or coal.

### Pre-operation Exergy, Emissions and Land Use for Coal

The total exergy required for mining coal, *β*_*coalmining*_, for a 1GW power plant over a 50 year lifetime is 6.69 × 10^4^ to 9.21 × 10^4^ GWhrs, without and with CCS, respectively, and is given by:

1 $${\beta }_{coalmining}={\mu }_{coalmining}\,\ast \,{M}_{coal}\,[{\rm{GWhrs}}]$$

where,

2 $${M}_{coal}=\frac{{\beta }_{out}}{{\eta }_{coalplant}\,\ast \,{\varepsilon }_{coal}}\,[{\rm{t}}]$$

where *β* is the exergy in GWhrs, *μ* is the specific exergy in GWh/t_coal_, M_coal_ is the total amount of coal required for combustion over the lifetime in tons, *β*_*out*_ is the desired electrical output of the plant over its lifetime in GWhrs, ŋ is efficiency and *ε* is the heat content of coal in GWh/ton.

The specific mining exergy for coal is 4.40 × 10^−4^ GWh_in_/t_coal_^39^ and the average heat content of coal consumed by electrical power plants in the U.S. is 6.54 × 10^−3^ GWh/ton^56^. The amount of coal required for the 1GW power plant without carbon capture technology during its lifetime ranges from 1.41 × 10^8^–1.77 × 10^8^ tons, with a realistic value of 1.52 × 10^8^ tons^22,29,36,42,56–59^ and with carbon capture it ranges from 1.85 × 10^8^–2.80 × 10^8^ tons with a realistic value of 2.10 × 10^8^ tons^22,29,42,56–61^. The efficiency of the plant drives the required exergy input and a review of the literature has found it to range from 32.50–40.60% with an average of 37.74% without carbon capture technology and 20.90–31.51% with an average of 27.40% with carbon capture technology^22,36,42,56,59,60,62^.

The total exergy required for transporting coal for a 1GW power plant over the 50 year lifetime, *β*_*coaltransport*_, is 3.17 × 10^4^ and 4.37 × 10^4^ GWhrs without and with CCS, respectively, and is given by:

3 $$\begin{array}{c}{\mu }_{coaltransport,truck}\\ {\mu }_{coaltransport,rail}+{\mu }_{coaltransport,marine}+\ast {M}_{coal}\\ {\beta }_{coaltransport}=\end{array}\,[{\rm{GWhrs}}]$$

where,

4 $$\mu =\vartheta \ast \phi \,[{\rm{GWh}}/{{\rm{t}}}_{{\rm{coal}}}]$$

where *ϑ* is the specific exergy in GWh/ton-km and *φ* is the specific distance in ton-km/t_coal_. Coal is transported for electrical generation via three main modes, rail, marine and truck, which account for 88%, 11% and 1%, respectively by ton-km^39^. Trains transport 1.04 ton-km/kg_coal_ at 1.81 × 10^−7^ GWh/ton-km, marine transports 130 ton-km/t_coal_ at 1.36 × 10^−7^ GWh/ton-km and trucks transport 10 ton-km/t_coal_ at 3.46 × 10^−7^ GWh/ton-km^39^.

The exergy required for construction, *β*_*coalconstruction*_, is 1.29 × 10^4^ GWhrs for a plant with and without CCS (the procurement of the additional equipment for compressing the CO_2_ is assumed to be negligible) and is calculated by:

5 $${\beta }_{coalconstruction}={\Sigma }({M}_{coalmaterials}\ast {\mu }_{coalmaterials})\,[{\rm{GWh}}]$$

where *M*_*coalmaterials*_ is the tonnage of individual materials^63^ and *μ*_*coalmaterials*_ is the specific exergy of the materials in GWh/t^39^. Total construction exergy is 9.4% of the total exergy required by upstream activities, detailed in Table 5.

**Table 5:** Construction exergy for large scale PV and coal electricity generation, each outputting 376 TWhrs over a 50 year lifetime.

	Material	Mass (tons)^63^	Specific Exergy Input (GJ/ton)^39^	Exergy Input (GWh)^39,63^
Coal Plant	Steel	6.22 × 10^4^	3.20	5.57 × 10^1^
	Aluminum	6.24 × 10^2^	3.87 × 10^1^	6.76
	Concrete	1.78 × 10^5^	8.16 × 10^−1^	4.07 × 10^1^
	Electricity	N/A	N/A	1.27 × 10^4^
	Oil	7.09 × 10^2^	4.08 × 10^1^	8.09
	Coal	1.43 × 10^4^	6.83 × 10^−6^	9.80 × 10^1^
	Total	2.56 × 10^5^		1.29 × 10^4^
PV Farm	Steel	1.04 × 10^3^	3.20	9.26 × 10^−1^
	Aluminum	4.00 × 10^1^	1.63 × 10^2^	1.82
	Concrete	5.00 × 10^2^	1.07	1.50 × 10^−1^
	Silicone	5.00 × 10^1^	5.71 × 10^3^	7.99 × 10^1^
	Glass	2.40 × 10^2^	1.71 × 10^1^	1.15
	Copper	1.08 × 10^2^	N/A	N/A
	Insulator	9.20 × 10^1^	N/A	N/A
	Electricity	N/A	N/A	1.70
	Oil	9.70 × 10^1^	4.08 × 10^1^	1.11
	Coal	2.90 × 10^1^	6.83 × 10^−6^	5.55 × 10^−8^
	Total	2.19 × 10^3^		8.68 × 10^1^

Total upstream greenhouse gas emissions for a 1GW coal plant, *π*_*coalupstream*_, are 3.92 × 10^7^ and 5.34 × 10^7^ ${{\rm{t}}}_{{{\rm{CO}}}_{2}}$_eq_ without and with CCS, respectively and are given by:

6 $$\begin{array}{rcl}{\pi }_{coalupstream} & = & ((({\alpha }_{coalmining}\ast {{\epsilon }}_{coalmining})+({\alpha }_{coaltransport}\ast {{\epsilon }}_{coaltransport}))\ast {M}_{coal})\\  &  & +({\alpha }_{coalconstruction}\ast {\beta }_{yealyoutput})\,[{{\rm{t}}}_{{{\rm{CO}}}_{2}{\rm{eq}}}]\end{array}$$

where *α* is the specific emissions in ${{\rm{t}}}_{{{\rm{CO}}}_{2}}$_eq_/t_coal_, ${\epsilon }$ is the percent contribution and *β*_*yealyoutput*_ is the exergy output per year from the operation phase (not including any downstream processes). The highest individual contributions come from mining and transportation. Specifically, mining emits 0.23 ${{\rm{t}}}_{{{\rm{CO}}}_{2}}$_eq_/t_coal_ (55% of total) and transport emits 0.19 ${{\rm{t}}}_{{{\rm{CO}}}_{2}}$_eq_/t_coal_ (45% of total), which totals 0.41 ${{\rm{t}}}_{{{\rm{CO}}}_{2}}$_eq_/t_coal_^39^. Furthermore, total upstream emissions are 55 ${{\rm{t}}}_{{{\rm{CO}}}_{2}}$_eq_/GWh_out_, resulting in 0.12 ${{\rm{t}}}_{{{\rm{CO}}}_{2}}$_eq_/t_coal_^43^. The average of these two values provides a total upstream emission factor of 0.27 ${{\rm{t}}}_{{{\rm{CO}}}_{2}}$_eq_/t_coal_. The yearly output is simply the lifetime output divided by 50, which is 7.5 TWhr/yr when the lifetime output is 376 TWhrs and 17.3 TWh/yr when the lifetime output is 866 TWhrs.

Construction of a large coal power plant emits 0.022 ${{\rm{kg}}}_{{{\rm{CO}}}_{2}}$_eq_/kWh-yr^63^, resulting in less than 1% of the total upstream emissions for the plant under study. More detailed information is provided in Table 6.

**Table 6:** GHG emission for the construction of a PV farm outputting 376 TWhrs over a 50 year lifetime.

Material	Mass (ton)^63^	Specific Emissions (${{\bf{t}}}_{{\bf{C}}{{\bf{O}}}_{{\bf{2}}}}$_eq_/ton)^39^	Emissions (${{\bf{t}}}_{{\bf{C}}{{\bf{O}}}_{{\bf{2}}}}$_eq_)^39,63^
Steel	1.04 × 10^3^	2.25 × 10^2^	2.33 × 10^5^
Aluminum	4.00 × 10^1^	8.80	3.52 × 10^2^
Concrete	5.00 × 10^2^	1.28 × 10^−1^	6.38 × 10^1^
Silicone	5.00 × 10^1^	3.30 × 10^2^	1.65 × 10^4^
Glass	2.40 × 10^2^	1.34	3.22 × 10^2^
Insulator	9.20 × 10^1^	N/A	N/A
Copper	1.08 × 10^2^	N/A	N/A
Electricity	N/A	N/A	1.38 × 10^3^
Oil	9.70 × 10^1^	3.88	3.77 × 10^2^
Coal	2.90 × 10^1^	1.60	4.64 × 10^1^
Total GHG Emissions	2.19 × 10^3^		2.52 × 10^5^

Mining emissions for the 1GW plant over its lifetime range from 1.14 × 10^7^–4.74 × 10^7^ ${{\rm{t}}}_{{{\rm{CO}}}_{2}}$_eq_ with a realistic value of 2.94 × 10^7^ ${{\rm{t}}}_{{{\rm{CO}}}_{2}}$_eq_ and 8.74 × 10^6^–3.44 × 10^7^ ${{\rm{t}}}_{{{\rm{CO}}}_{2}}$_eq_ with a realistic value of 2.16 × 10^7^ ${{\rm{t}}}_{{{\rm{CO}}}_{2}}$_eq_ for a plant with and without capture, respectively^39,43^.

Transport emissions for the 1GW plant over its lifetime range from 9.30 × 10^6^–3.88 × 10^7^ ${{\rm{t}}}_{{{\rm{CO}}}_{2}}$_eq_ with a realistic value of 2.4 × 10^7^ ${{\rm{t}}}_{{{\rm{CO}}}_{2}}$_eq_ and 7.15 × 10^6^–2.81 × 10^7^ ${{\rm{t}}}_{{{\rm{CO}}}_{2}}$_eq_ with a realistic value of 1.76 × 10^7^ ${{\rm{t}}}_{{{\rm{CO}}}_{2}}$_eq_ for a plant with and without capture, respectively^39,43^.

Land transformation for upstream activities for a 1GW coal plant, *A*_*coalupstream*_, are 17.8 kha and 22.7 kha without and with CCS, respectively and calculated by:

7 $${A}_{coalupstream}=({\tau }_{coalmining}+{\tau }_{coaltransport})\ast {\beta }_{out}\,[{\rm{kha}}]$$

where *τ*_*coal*_ is the specific land transformation in ha/t_coal_. In the U.S., there are 195 kha of land leased for coal mining, but only 140 kha of the land actively being used^64^ with 8.97 × 10^8^ tons of coal mined each year^40^ giving an average of 1.52 × 10^−4^ ha/t_coal_. Surface mining transforms 90–1,820 m^2^/kt with a realistic value of 300 m^2^/kt and underground mining transforms 4.5–1,110 m^2^/kt with a realistic of 30 m^2^/kton^48^. Given that on average 70% of the coal mined in the U.S. is from surface mining and 30% from underground mining^48^, the average of these various values was taken to provide a realistic value of 8.70 × 10^−5^ ha/t_coal_.

Specific land transformed by rail infrastructure ranges from 30 m^2^/GWh in the east to 80 m^2^/GWh in the west^48^. Given that 88% of coal shipped to electrical power plants is by rail^65^ and another 11% by water^39^ it is assumed the land transformed by rail is representative of the total land transformation. 55% of the coal is mined in the west and 45% in the east^66^ so a realistic value was assumed to be 5.75 × 10^−3^ ha/GWh. When multiplied by the ratio of *β*_*out*_ over t_coal_ consumed by the plant with and without carbon capture over its lifetime it equates to 1.42 × 10^−5^ ha/t_coal_ and 1.03 × 10^−5^ ha/t_coal_, respectively. Each range from 1.22 × 10^−5^–1.53 × 10^−5^ ha/t_coal_ and 7.72 × 10^−6^–1.17 × 10^−5^ ha/t_coal_, respectively.

The land transformed by the upstream activities for the construction of the coal power plant is not included in the scope of this analysis rendering all values for coal conservative over the entire life cycle.

### Pre-operation Exergy, Emissions and Land Use for PV

The exergy required for upstream activities, *β*_*PVupstream*_, is 2.00 × 10^4^ GWhrs for PVs1 and 4.60 × 10^4^ GWhrs for PVs2 and is calculated by:

8 $${\beta }_{PVupstream}=({\dot{{\rm{t}}}}_{EPB}\ast {\beta }_{yearlyoutput})+{\beta }_{PVconstruction}\,[{\rm{GWhrs}}]$$

where ${\dot{{\rm{t}}}}_{EPB}$ is the energy payback time in years, which ranges from 1.7–5.5 years, with an average of 2.7 years^49,50^. This was multiplied by the yearly output to determine the upstream exergy input of 5.3 × 10^−2^ GWh_in_/GWh_out_. Individually, the energy contribution from the modules and BOS are 63% and 37% of the total, respectively^67^. The construction exergy was calculated by multiplying the tonnage of material by the upstream exergy for each material^39,63^, more detailed data is provided in Table 5.

The upstream GHG emissions, *π*_*PVupstream*_, are 8.92 × 10^6^ ${{\rm{t}}}_{{{\rm{CO}}}_{2}}$_eq_ and 1.70 × 10^7^ ${{\rm{t}}}_{{{\rm{CO}}}_{2}}$_eq_ for PVs1 and PVs2, respectively and is calculated with:

9 $${\pi }_{PVupstream}=(\frac{{\alpha }_{PVupstream}\ast {\beta }_{out}}{2})+{\pi }_{PVconstruction}\,[{{\rm{t}}}_{{{\rm{CO}}}_{2}{\rm{eq}}}]$$

where *α*_*PV*_ is the specific GHG emissions in ${{\rm{t}}}_{{{\rm{CO}}}_{2}}$_eq_/GWh_out_ in its life cycle, which range from 8.74–187 ${{\rm{t}}}_{{{\rm{CO}}}_{2}}$_eq_/GWh_out_, with a realistic value of 46.98 ${{\rm{t}}}_{{{\rm{CO}}}_{2}}$_eq_/GWh_out_^38,49,50,68,69^. The individual contributions of modules and BOS to total emissions are 38.9% and 61.1%, respectively^67^. The emissions from construction are detailed in Table 6. LCAs typically assumed a lifetime of 20–30 years. The values given in this paper assume that a negligible amount of greenhouse gases are emitted and after 25 years of operation, and a negligible amount of GHG’s are emitted.

Land transformation from upstream activities, *A*_*PVupstream*_, are 0.58 kha and 1.12 kha for PVs1 and PVs2, respectively and is calculated with:

10 $${A}_{PVupstream}=\frac{({\tau }_{PVmodules}+{\tau }_{PVBOS})\ast {\beta }_{out}}{2}\,[{\rm{kha}}]$$

where *τ*_*PV*_ is the specific land transformation in ha/GWh_out_. Land transformation for upstream activity is 1.84 × 10^−3^ ha/GWh_out_ and 7.5 × 10^−4^ ha/GWh_out_ for modules and BOS, respectively^48^. The upstream land transformation for materials and processes specific to construction of the PV farm were not included so total values can be considered conservative.

### Operation Exergy, Emissions and Land Use for Coal

The exergy into the coal plant during operation is comprised entirely from the latent energy in the coal. The exergy inputs required, *β*_*coaloperation*_, are 9.95 × 10^5^ and 1.37 × 10^6^ GWhrs without and with CCS, respectively and are calculated by:

11 $${\beta }_{coaloperation}=\frac{{\beta }_{out}}{{{\rm{n}}}_{plant}}\,[{\rm{GWhrs}}]$$

The efficiency of a state-of-the-art plant without CCS ranges from 32.5–40.6% with a realistic value of 37.74%, requiring an input range from 9.25 × 10^5^–1.16 × 10^6^ GWh with a realistic value of 9.95 × 10^5^ GWh^22,29,36,42,57,60,62^. With various forms of CCS, the efficiency ranges from 20.90–31.51% with a realistic value of 27.4% requiring an exergy input range from 1.21 × 10^6^–1.83 × 10^6^ GWh with a realistic value of 1.637 × 10^6^ GWh^22,29,42,57,59–61^. Thus, carbon capture technology necessitates 37.74% more coal, which creates additional GHG emissions. The realistic capture in this study is taken as 82.2%.

The carbon capture efficiency ranges from 81–91% capture of total emissions^22,29,41,42,57,59–61^. The most common and technologically mature method of carbon capture at the plant is post-combustion capture using MEA. Several other carbon capture processes were included in the purview of the study and are shown in Table 7. The efficiency drop due to CCS comes from the high energy intensity of the carbon capture process. Large-scale MEA processes can consume 92–119 MW_el_ and an additional 0.72–1.74 MW_th_/MW_eloutput_. This results in and average of 0.11 GW_el_ and 0.99 GW_th_ for a ~1GW power plant^41^. These values are conservative because the carbon capture percentage in the study was 60–65%. The average energy efficiency of a state of the art coal plant in the U.S. is used after having identified the energy efficiency for the top 10% of the fleet^36^.

**Table 7:** Range of pulverized coal plant efficiencies equipped with various forms of carbon capture.

Efficiencies	Min (%)	Max (%)	Realistic (%)
MEA	26.24^41^	29.90^57^	28.03^22,29,41,42,57^
Membrane*	27.23^60^	31.67^59^	29.77^59,60,99^
Ammonia			27.90^42^
Other	20.90^29^	33.36^29^	26.77^29^

*Membranes are either 2-stage or 2-stage with a 1 or 2 stage air sweep.

The GHG emissions to the atmosphere during operation, *π*_*coaloperation*_, are 3.38 × 10^8^ and 6.07 × 10^7^ ${{\rm{t}}}_{{{\rm{CO}}}_{2}}$_eq_ without and with CCS, respectively and are calculated by:

12 $${\pi }_{coaloperation}={\alpha }_{coaloperation}\ast {\beta }_{out}\,[{{\rm{t}}}_{{{\rm{CO}}}_{2}{\rm{eq}}}]$$

where the specific GHG emissions from coal plants without carbon capture range from 807–1100 ${{\rm{t}}}_{{{\rm{CO}}}_{2}}$_eq_/GWh, with a realistic value of 900 ${{\rm{t}}}_{{{\rm{CO}}}_{2}}$_eq_/GWh^38,44,45,58^. Emissions from plants with capture range from 124–203 ${{\rm{t}}}_{{{\rm{CO}}}_{2}}$_eq_/GWh, with a realistic value of 160 ${{\rm{t}}}_{{{\rm{CO}}}_{2}}$_eq_/GWh^22,44,45,58^.

Land transformation caused by the plant itself, *A*_*coaloperation*_, is 202 ha and calculated by:

13 $${A}_{coaloperation}={\tau }_{coaloperation}\ast {\beta }_{out}\,[{\rm{kha}}]$$

where the specific land transformation ranges from 6.0 × 10^−4^–3.3 × 10^−3^ ha/GWh_out_, with an average of 9.0 × 10^−4^ ha/GWh_out_^48^.

### Operation Exergy, Emissions and Land Use for PV

In the operation phase, the solar irradiation accounts for the entirety of the exergy input, *β*_*PVoperation*_, totaling 3.50 × 10^6^ and 8.05 × 10^6^ GWhrs for PVs1 and PVs2, respectively and is calculated by:

14 $${\beta }_{PVoperation}={\Sigma }(\frac{{\beta }_{yearlyoutput}}{{{\rm{\eta }}}_{PVyearly}})\,[{\rm{GWhrs}}]$$

where,

15 $${{\rm{\eta }}}_{PVyearly}={{\rm{\eta }}}_{0}\ast {(1-d)}^{n}\,[ \% ]$$

where ŋ_0_ is the initial exergy efficiency of the PV system, *d* is the degradation rate in %/yr and *n* is the years of operation. The exergetic efficiency of PV was found to range from 7.8–16.1%, with a realistic value being 12.1%^51,52,70–72^. The degradation rate ranged from 0.35–0.8%/yr with a realistic average of 0.49%/yr^37,73^. System Advisor Model (SAM) from the National Renewable Energy Laboratory (NREL) was employed to ensure the accuracy of values from the calculations above^74^.

The GHG emissions released to the atmosphere during operation, *π*_*PVoperation*_, are 8.69 × 10^4^ and 2.01 × 10^5^ ${{\rm{t}}}_{{{\rm{CO}}}_{2}}$_eq_ for PVs1 and PVs2, respectively, and calculated by:

16 $${\pi }_{PVoperation}=\frac{{\alpha }_{PVoperation}\ast {\beta }_{out}}{2}\,[{{\rm{t}}}_{{{\rm{CO}}}_{2}{\rm{eq}}}]$$

A range of 0–46.3 ${{\rm{t}}}_{{{\rm{CO}}}_{2}}$_eq_/GWh_out_^38^ are emitted during installation of a PV farm. The worst case assumes locating a PV farm in a heavily forested area with CO_2_ emissions from loss of forest sequestration, soil respiration and oxidation of cut biomass. An assumption of 0.46 ${{\rm{t}}}_{{{\rm{CO}}}_{2}}$/GWh (1%) from deforestation was employed for this study as forests are not typically clear cut for PV farms.

Land transformation due to the PV farm, *A*_*PVoperation*_, is 3.94 and 9.51 kha and 9.22 and 26.46 kha for PVs1 and PVs2, respectively and are calculated as the average of two approaches:

17 $${A}_{PVoperation}={\tau }_{PVoperation}\ast {C}_{NP}\,[{\rm{kha}}]$$

18 $${A}_{PVoperation}={\tau }_{PVoperation}\ast {\beta }_{out}\,[{\rm{kha}}]$$

where ${\tau }_{PV}$ is the specific land transformation in ha/GW and *C*_*NP*_ is the nameplate capacity. The PV farms themselves ranges from 2.02–3.23 kha/GW^38^, while a review of three of the largest PV farms in the United States (Solar Star, Mount Signal and California Valley) reveals that they are 2.25, 3.89 and 5.20 kha/GW, respectively, giving an average of 3.32 kha/GW^75–77^. Land transformation for the modules and BOS combined range from 1.64 × 10^−2^ ha/GWh_out_ to 4.62 × 10^−2^ ha/GWh_out_, with a realistic value of 3.59 × 10^−2^ ha/GWh_out_^48^. These were multiplied by nameplate capacity or lifetime exergy output and then averaged together to determine the final values. It should be noted here that the PV farms are best suited from an environmental standpoint or barren land or existing man-made structures (e.g. rooftops, sound barriers, parking lot awnings, etc.) and should be used before fertile land is used because of the negative impacts on food price and availability.

### Downstream Exergy, Emissions and Land Use for Coal

The exergy input from solar irradiation for bio-sequestration of GHG emission from coal without CCS, *β*_*coalbio*_, is 2.57 × 10^8^ GWhrs and calculated by:

19 $${\beta }_{coalbio}=G\ast N\ast {A}_{coalbio}\,[{\rm{GWhrs}}]$$

where *G* is the average U.S. solar incidence of 15,000 GWh/ha * yr^78^, *N* is the number of years over its lifetime and *A*_*coalbio*_ is the land transformation required by the switchgrass for upstream and operation activities without CCS in hectares, explained in equation 32 below.

The exergy input for bio-sequestration of GHG emissions from coal with CCS into a saline aquifer, *β*_*coalCCS*_, is 8.14 × 10^7^ GWhrs and calculated with:

20 $${\beta }_{coalCCS}={\beta }_{coalCCSbio}+{\beta }_{C{O}_{2}cond}\,[{\rm{GWhrs}}]$$

21 $${\beta }_{coalCCSbio}=G\ast N\ast {A}_{coalCCSbio}\,[{\rm{GWhrs}}]$$

22 $${\beta }_{C{O}_{2}cond}={\mu }_{C{O}_{2}cond}\ast \gamma \,[{\rm{GWhrs}}]$$

where *A*_*coalCCSbio*_ is the land transformation required by the switchgrass for upstream, operation and downstream activities with CCS into a saline aquifer in hectares explained in equation 33 below, ${\mu }_{C{O}_{2}cond}$ is the specific exergy required to condition the CO_2_ (compress and transport) after its been separated and measured in GWh/${{\rm{t}}}_{{{\rm{CO}}}_{2}}$ and *γ* is the total CO_2_ captured in ${{\rm{t}}}_{{{\rm{CO}}}_{2}}$. CO_2_ is typically transported via pipeline in a supercritical state, between 8.6–15.3 MPa^43^. The specific energy required to compress CO_2_ is between 112–119 kWh/${{\rm{t}}}_{{{\rm{CO}}}_{2}}$, realistically being 116 kWh/${{\rm{t}}}_{{{\rm{CO}}}_{2}}$^41,78^. The pipelines have been found to lose between 4–50 kPa per 100 km^79^, thus requiring 6.5 kWh/${{\rm{t}}}_{{{\rm{CO}}}_{2}}$ to boost the pressure for longer transport^43^ but the assumption in this study is that no pressure boosters are required. The average distance for CO_2_ to travel for sequestration purposes is 190.5 km^32^ and the Weyburn case demonstrates that CO_2_ can be transported 330 km without additional boosting energy^78^. The total CO_2eq_ captured is the difference between GHG emissions from a coal without CCS and a coal plant with CCS, which are 3.38 × 10^8^ ${{\rm{t}}}_{{{\rm{CO}}}_{2}}$_eq_ and 6.02 × 10^7^ ${{\rm{t}}}_{{{\rm{CO}}}_{2}}$_eq_, respectively.

The exergy input for bio-sequestration of GHG emission from coal with CCS for EOR,*β*_*coalEOR*_, is 2.13 × 10^8^ GWhrs and calculated with:

23 $${\beta }_{coalEOR}={\beta }_{coalbio}+{\beta }_{coalCCSbio}+{\beta }_{oilextraction}+{\beta }_{oiltransport}+{\beta }_{oilrefine}\,[{\rm{GWhrs}}]$$

where,

24 $${\beta }_{oilextraction}={\mu }_{oilextract}\ast {M}_{oil}\,[{\rm{GWhrs}}]$$

25 $${\beta }_{oiltransport}={\mu }_{oiltransport}\ast {M}_{oil}\ast {D}_{oil}\,[{\rm{GWhrs}}]$$

26where,

27 $${M}_{oil}=\gamma \ast {\theta }_{oil}\,[{\rm{t}}]$$

and where *μ*_*oilextract*_ is the specific exergy required to pump the oil from the reservoir in GWh/t_oil_, *M*_*oil*_ is the amount of additional oil extracted with the EOR process in t_oil_, ${\mu }_{oiltransport}$ is the specific exergy to transport the oil to the refinery in GWh/ton-km_oil_, *D*_*oil*_ is the average distance oil travels to the refinery in km, ŋ_*refinery*_ is the efficiency of the refinery, ${{\epsilon }}_{oil}$ is the energy content of crude oil and *θ*_*oil*_ is the specific oil production from the EOR process in t_oil_/${{\rm{t}}}_{{{\rm{CO}}}_{2}}$.

For enhanced oil recovery, it takes 4.40 × 10^−5^–1.38 × 10^−4^ GWh/t_oil_, with a realistic value of 7.40 × 10^−5^ GWh/t_oil_ to extract crude oil^61,80^. The exergy required for recycling and re-injecting the CO_2_ continuously ranges between 3.21–9.00 kWh/${{\rm{t}}}_{{{\rm{CO}}}_{2}}$_injected_, with a realistic value of 6.10 kWh/${{\rm{t}}}_{{{\rm{CO}}}_{2}}$_injected_^43,80^. The exergy required for recycling the CO_2_ is captured under the extraction exergy.

An additional exergetic input of 8.15 × 10^8^ GWh/t_oil_ is needed to transport it to a refinery and the average distance crude oil travels to a refinery is 1200 km^43^.

A typical refinery operates at 90.1% efficiency^80^ and approximately 93% of this turns into combustible products^43^. The crude oil was assumed to have a heat content of 1.17 × 10^−2^ GWh/ton^78^ and all the refined product to have a heat content of 1.14 × 10^−2^ GWh/ton^39^. The exergy required to transport the refined product is considered negligible^43^.

The specific tonnage of oil produced from EOR ranges from 0.18 to 0.89 t_oil_/${{\rm{t}}}_{{{\rm{CO}}}_{2}}$_injected_ with an average of 0.43 t_oil_/${{\rm{t}}}_{{{\rm{CO}}}_{2}}$_injected_^34,46,80–82^.

Equations 28–32 calculate the GHG emissions of coal without CCS, *π*_*coalbio*_, coal with CCS into a saline aquifer, *π*_*coalCCS*_, and coal with CCS for EOR, *π*_*coalEOR*_, which are 3.77 × 10^8^, 1.18 × 10^8^ and 3.11 × 10^8^ ${{\rm{t}}}_{{{\rm{CO}}}_{2}}$_eq_, respectively.

28 $${\pi }_{coalbio}={\pi }_{coalupstream}+{\pi }_{coaloperation}\,[{{\rm{t}}}_{{{\rm{CO}}}_{2}{\rm{eq}}}]$$

29 $${\pi }_{coalCCS}={\pi }_{coalupstream}+({\pi }_{coaloperation}\ast (1-\gamma ))+{\pi }_{leak}\,[{{\rm{t}}}_{{{\rm{CO}}}_{2}{\rm{eq}}}]$$

30 $${\pi }_{coalEOR}={\pi }_{coalCCS}+{\pi }_{EOR}\,[{{\rm{t}}}_{{{\rm{CO}}}_{2}{\rm{eq}}}]$$

where,

31 $$\begin{array}{c}{\rho }_{reservoir}\ast N\ast \gamma \\ {\alpha }_{C{O}_{2}transport}\ast {D}_{C{O}_{2}}\\ {\pi }_{leak}=\end{array}\,[{{\rm{t}}}_{{{\rm{CO}}}_{2}{\rm{eq}}}]$$

32 $${\pi }_{EOR}=[({\rho }_{extraction})+[({\theta }_{transport}+{\theta }_{refine}+{\theta }_{combust})\ast {\varepsilon }_{oil}]]\ast \gamma \,[{{\rm{t}}}_{{{\rm{CO}}}_{2}{\rm{eq}}}]$$

where *ρ*_*reservoir*_ is the leakage rate from the oil and gas reservoir in %/yr, *α*_*CO*2*transport*_ is the specific emissions from the pipeline transport of CO_2_ in ${{\rm{t}}}_{{{\rm{CO}}}_{2}}$/km, ${D}_{C{O}_{2}}$ is the distance CO_2_ travels in the pipe from the plant to the reservoir in km, *ρ*_*extraction*_ is the CO_2_ released to the atmosphere during the recycling and re-injection and *θ* is the specific emissions in ${{\rm{t}}}_{{{\rm{CO}}}_{2}}$/bbl, where bbl is short for a barrel of oil and 7.33 bbl equate to one metric ton of crude oil.

The downstream processes of EOR emit significant amounts of greenhouse gas. Separating and recycling the CO_2_ for re-injection is important to curtail emissions during EOR. Alternating floods of water and CO_2_ gas are injected into oil deposits to increase oil production. For EOR optimized for carbon sequestration, it can take months for the CO_2_ to start being extracted with the crude oil and will continue to be extracted for years after flooding has stopped^46^. During crude oil extraction, 13.7% of the total injected CO_2eq_ is lost to the atmosphere when assuming that CO_2_ is injected for 10 years and then recycled for another 10 years. 11% of these losses come from recycling, 38% from venting CO_2_ and 42% from venting CH_4_^46^. Transport of crude oil to the refinery emits 4 × 10^−3^ ${{\rm{t}}}_{{{\rm{CO}}}_{2}}$_eq_/bbl, refining the crude emits 3 × 10^−2^ ${{\rm{t}}}_{{{\rm{CO}}}_{2}}$_eq_/bbl and combusting the refined product emits 0.43 ${{\rm{t}}}_{{{\rm{CO}}}_{2}}$_eq_/bbl^46^. The energy content of crude oil is 41.9 GJ/ton^80^. Transportation of the refined product is considered negligible^43^.

The target for leakage from geological storage, like that used in saline aquifers and EOR, should be between 1 × 10^−2^–1 × 10^−1^%/yr or 1 × 10^−3^–1 × 10^−2^%/yr^31,83^, so a realistic value of 2.75 × 10^−2^%/yr is used. There are over 4,500 km of CO_2_ pipelines^83^ which emit 4.64 × 10^7^ ${{\rm{t}}}_{{{\rm{CO}}}_{2}}$_eq_ each year^10^, resulting in emissions of 1.03 × 10^4^ ${{\rm{t}}}_{{{\rm{CO}}}_{2}}$_eq_/km and the average distance the CO_2_ is pumped is 190.5 km^32^.

The land transformation required for bio-sequestration for coal without CCS, *A*_*coalbio*_, coal with CCS into a saline aquifer, *A*_*coalCCS*_, and coal with CCS for EOR, *A*_*coalEOR*_, is 343, 109 and 284 kha, respectively and calculated using:

33 $${A}_{coalbio}=\frac{{\pi }_{coalbio}\ast \sigma }{\omega \ast N}\,[{\rm{kha}}]$$

34 $${A}_{coalCCS}=\frac{({\pi }_{coalbio}+{\pi }_{leak})\ast \sigma }{\omega \ast N}\,[{\rm{kha}}]$$

35 $${A}_{coalEOR}=\frac{({\pi }_{coalbio}+{\pi }_{leak}+{\pi }_{EOR})\ast \sigma }{\omega \ast N}\,[{\rm{kha}}]$$

where *σ* is the molar ratio of carbon to CO_2_, which is $(\frac{12}{44})$ and *ω* is the rate of carbon uptake by switchgrass, which is 6 t_C_/ha-yr^84^. The scenario without CCS does not have any GHG emissions from leakage or EOR and the scenario with CCS into a saline aquifer does not have any GHG emissions from EOR. The pipelines used to transport CO_2_ are considered to be buried and hence have a negligible amount of land transformation.

### Downstream Exergy, Emissions and Land Use for PV

Solar incidence on the land required for sequestration accounts for the total exergy into this phase of the analysis. The exergy inputs, *β*_*PVbio*_, are 1.29 × 10^7^ and 2.59 × 10^7^ GWh for PVs1 and PVs2, respectively are calculated with:

36 $${\beta }_{PVbio}=G\ast N\ast {A}_{PVbio}\,[{\rm{GWhrs}}]$$

where,

37 $${A}_{PVbio}=\frac{({\alpha }_{PVupstream}+{\alpha }_{PVoperation})\ast {\beta }_{yearlyoutput}\ast \sigma }{\omega }\,[{\rm{kha}}]$$

Switchgrass offers the best carbon sequestration potential of 6 t_C_/ha-yr^84^ and is assumed to sequester the CO_2eq_ released by the implementation of the PV farm. The sequestration potentials of various biomass can be seen in Table 8. It has been shown to sequester steadily for over 50 years with little maintenance^85^.

**Table 8:** Carbon uptake rates of various types of biomass.

Biomass Type	Value (tC/ha * yr)
Switchgrass	6.0^84^
Poplar	5.4^84^
Willow	4.3^84^
Woody Tissue	3.8^25^
Average US Forest	0.7^26^

The total emissions from PV to be sequestered by biomass, *π*_*PVbio*_, are 9.01 × 10^6^ and 1.72 × 10^7^ ${{\rm{t}}}_{{{\rm{CO}}}_{2}}$_eq_ for PVs1 and PVs2 respectively, over the 50 year lifetime and are calculated by:

38 $${\pi }_{PVbio}={\pi }_{upstream}+{\pi }_{operation}\,[{{\rm{t}}}_{{{\rm{CO}}}_{2}{\rm{eq}}}])$$

It should be noted that the values for PV can be improved further in the future as widespread PV recycling becomes widespread^86,87^. To date the vast majority of PV is still operational, however, in the future recycling of PV will become significantly more important. Advanced recycling can reduce the embodied energy of PV on the manufacturing end by enabling industrial symbiosis^88–90^. This transfer to waste products back into the wealth created by PV electricity generation can directly benefit the circular economy^91^.

Lastly, it should be pointed out that all more efficient dual uses of land were not considered (e.g. mounting PV on the rooftops of CCS facilities or using the surface area in-between rows of PV for agricultural farming (agrivoltaics^92–95^).

## Conclusions

The growth and maturation of photovoltaic technology has enabled it to provide large-scale electricity generation and supplant existing large-scale coal generation. Both solar and coal technologies have the capacity to be climate neutral using bio-sequestration and CCS. The additional land area required to bio-sequester coal-fired electricity in the U.S. is physically impossible in some cases and not realistic in the best case, where CCS and EOR do improve coal performance. Even with the best available technologies the use of coal to provide climate-neutral power cannot be justified because the potential for far more effective use of land with PV. This study showed that solar photovoltaic technology is a far superior use of land for climate neutral electricity generation than any technology coupled to coal.

Recent advances have made CCS more feasible, and in conjunction with EOR more practical. However, the process of EOR only sequesters 28% of the CO_2_ injected due to subsequent downstream emissions. But, when comparing coal emissions on a per GWh_electric output_ basis, a plant with CCS for EOR is only slightly worse to a plant with CCS into saline aquifers. Largely because the combustion of oil is less polluting than the combustion of coal, which mitigates its inherent emissions.

The results of this study have shown that CCS is unable to make climate-neutral coal competitive with climate-neutral PV in average solar conditions. Climate-neutral photovoltaic farms are a better option than climate neutral coal from an exergy, GHG emissions and land transformation perspective, by several orders of magnitude each. Future work is needed to carefully consider the cost benefit analysis of policies to support climate-neutral electricity generation. Research and policy promoting rapid deployment in photovoltaic technology offers more promising solutions to combat climate change than continued research into advanced coal and CCS.
